# Compound motion decoding based on sEMG consisting of gestures, wrist angles, and strength

**DOI:** 10.3389/fnbot.2022.979949

**Published:** 2022-11-10

**Authors:** Xiaodong Zhang, Zhufeng Lu, Chen Fan, Yachun Wang, Teng Zhang, Hanzhe Li, Qing Tao

**Affiliations:** ^1^School of Mechanical Engineering, Xi'an Jiaotong University, Xi'an, China; ^2^Shaanxi Key Laboratory of Intelligent Robot, Xi'an Jiaotong University, Xi'an, China; ^3^Department of Mechanical Engineering, Xi'an Jiaotong University City College, Xi'an, China; ^4^School of Mechanical Engineering, Xinjiang University, Wulumuqi, China

**Keywords:** surface EMG, compound motion decoding, deep learning, machine learning, myoelectric prosthesis

## Abstract

This study aimed to highlight the demand for upper limb compound motion decoding to provide a more diversified and flexible operation for the electromyographic hand. In total, 60 compound motions were selected, which were combined with four gestures, five wrist angles, and three strength levels. Both deep learning methods and machine learning classifiers were compared to analyze the decoding performance. For deep learning, three structures and two ways of label encoding were assessed for their training processes and accuracies; for machine learning, 24 classifiers, seven features, and a combination of classifier chains were analyzed. Results show that for this relatively small sample multi-target surface electromyography (sEMG) classification, feature combination (mean absolute value, root mean square, variance, 4th-autoregressive coefficient, wavelength, zero crossings, and slope signal change) with Support Vector Machine (quadric kernel) outstood because of its high accuracy, short training process, less computation cost, and stability (*p* < 0.05). The decoding result achieved an average test accuracy of 98.42 ± 1.71% with 150 ms sEMG. The average accuracy for separate gestures, wrist angles, and strength levels were 99.35 ± 0.67%, 99.34 ± 0.88%, and 99.04 ± 1.16%. Among all 60 motions, 58 showed a test accuracy greater than 95%, and one part was equal to 100%.

## Introduction

Surface electromyography (sEMG) is a bioelectric signal naturally produced during the neural activation of muscles (Vredenbregt and Rau, 1973). Through the mapping relationship between the activation degree and the position of muscles, sEMG contains the movement intention of the human body. It has been considered to be one of the modalities of human-machine interface (HMI) in the context of human-centered robotics (Zhang et al., 2015). Compared with other bioelectric signals, such as electroencephalograms, sEMG shows stronger controllability, more decoding patterns, and higher stability. As one popular representation of human intention, sEMG gets its widest application in controlling a myoelectric hand (De Luca, 1997), an exoskeleton (Kiguchi and Hayashi, 2012), and so on.

To realize the sEMG-based control, a number of research studies focused on gesture decoding were carried out first. As early as the 1970s, Taylor D began using the sEMG collected by multi-electrode arrays to control upper limb prostheses (Wirta et al., 1978). In 2007, Chu et al. (2007) achieved an average accuracy of 97.4% in nine kinds of hand motion decoding with four surface electrodes (Chu et al., 2007). In 2016, Adenike realized the decoding of 19 classes, including hand grasps and individual finger motions, and achieved an accuracy of 96% for non-amputees (Adewuyi et al., 2016). To reduce the individual differences, Xue proposed a novel user-independent framework on 13 gesture decoding with an accuracy of 78.15% in 2021, which combined the canonical correlation analysis and optimal transport (Boschmann et al., 2013).

Gesture decoding above took the lead in ensuring the basic grasping function. For a human hand, the complete movement relies not only on the fingers and the palm but also on the cooperation of the wrist and elbow. Beyond basic EMG decoding of gestures, researchers have paid attention to the wrist, the elbow, and the compound motions. In 2010, Zeeshan used the forearm sEMG to classify 19 wrist torques, which showed an 88% accuracy (Khokhar et al., 2010). In 2019, Zhang et al. (2019) proposed a novel preprocessing method for joint force estimation with high-density sEMG. In 2021, Xiang Chen reported a convolutional neural network with a transfer learning strategy in decoding 30 hand gestures involving various states of the finger, elbow, and wrist, which achieved an accuracy of 92.13% with high-density sEMG (Chen et al., 2021). Other studies, such as Zhang et al. (2011); Lu et al. (2014); McIntosh et al. (2016), and so on, have made gesture classification with wrist coupling by combining sEMG with additional sensors.

The sEMG-based decoding of joint movement allows for a more versatile application. Meanwhile, considering the spatio-temporal difference, the decoding of compound actions also guarantees the stability of gesture decoding in multiple poses of the upper limbs. A more practical way to apply the decoded targets to the control of the myoelectric hand is by combining machine intelligence with human intention. By fully using the closed-loop control and the sensory feedback, the decoded target can be viewed as merely enabling a flag, relying upon the prosthesis to complete the blind grasp. In 2011, Hao Dang proposed a stable robotic grasping method based on tactile feedback and hand kinematics, which can further be applied to the blind grasping of the myoelectric hand (Dang et al., 2011). In 2016, Xiong reported the implementation of an anthropomorphic hand for replicating human grasping functions, which realized the blind grasp automatically and was further endued with myoelectric control (Xiong et al., 2016). In 2020, Mayer et al. (2020) reported a closed-loop control method based on tactile feedback to ensure the grasping of the myoelectric hand. Meanwhile, leading commercial prostheses such as the Michelangelo prosthetic hand by Ottobock© (Hashim et al., 2017) and the i-Limb by Össur© (van der Niet et al., 2013) provide customers with EMG-based solutions combined with intelligence control to ensure better practice for daily usage.

By properly combining human intention and machine intelligence, grasping the myoelectric can be more stable and realistic than relying on real-time sEMG-decoding alone. In addition to the grip, through daily observation, we have noticed that different control purposes can exist within the same gesture, such as “grip an egg” vs. “crush an egg.” The expression of these detailed purposes has been mostly neglected in the design of the myoelectric hand. Most of the research has focused on one purpose, possibly firmly grasping, to carry out the closed-loop control.

For a fixed gesture, different control purposes (such as griping vs. crushing) mainly correspond to different strength levels. Considering the controllability and the measurement in research, we mapped the strength level to different load levels. In addition to the distinction of control purpose, to ensure the stability of gesture decoding at different wrist angles, there was also a demand for composite motion decoding. In this paper, focusing on flexible myoelectric control and the control purposes of switching, the decoding of compound motions was proposed. These compound motions consisted of the product of gestures, wrist angles, and strength levels, allowing for simultaneous control of the gesture and wrist, as well as switching control purposes. The Materials and Methods section describes the selection basis for compound actions, the experimental setup and data segment, and the various methods adopted for compound motion decoding and performance comparison. The Result section reports the detailed result. The Discussion and the Conclusion sections state the discussion and conclusion separately.

## Materials and methods

### Demands of compound motion decoding

#### Control strategy of the myoelectric hand

In reality, the myoelectric hand control merely relies on sEMG-decoding, which is obviously unstable due to the inevitable online misrecognition. Even a tiny misrecognition can result in the failure of a whole task. Moreover, the misoperation caused by the misrecognition may decrease the user's faith in the myoelectric system, resulting in a worse operating state. Therefore, a good combination of human intention and machine intelligence is more realistic to ensure practical controllability.

When reaching into a bag, it is intuitive and straightforward for a natural human hand to grasp objects without any pre-existing geometric or visual information (Dang et al., 2011). To implement the same function in the anthropomorphic hand, blind grasping, also known as the hot spot technique, has been studied by many research groups worldwide. As human beings, the grasping gesture was gradually formed according to the tactile perception of the object's surface. Depending on the sensory-feedback closed-loop control, the myoelectric hand can share the same grasping strategy as human hands. By adopting blind grasping to grip firmly, the gesture of the myoelectric hand can automatically be detected instead of being defined by the sEMG-decoding. Compared with sEMG-dependent gesture decoding, such a scheme has higher stability and practicability. Thus, with this strategy, the sEMG target can be viewed as an enabling flag rather than a real-time control command for firmly grasping. It significantly reduced the grasping gestures that needed to be decoded *via* sEMG. According to tactile-based blind grasping, the robust control law tends to use all known fingers to perceive unknown objects, thus completing the power grasp (Shaw-Cortez et al., 2019). By selecting one sEMG enabling flag corresponding to the blind power grasping with all fingers (Shaw-Cortez et al., 2019), another sEMG-based detailed decoding can be left to precision grasp gesture (such as pinch), gestures with specific usage (such as poke) and other sign languages.

During grasping, since there can be multiple purposes under one same gesture (such as griping vs. crushing), the specification of control purpose *via* sEMG is necessary. Under one fixed gesture, different purposes mainly correspond to different levels of muscle strength and can be mapped to multiple control logics of the anthropomorphic hand. In the classic, such myoelectric control directly associated with the proportion of strength is regarded as the direct control (DC) approach (Mereu et al., 2021). By going a step further to distinguish the strength level under different postures, a more diverse purpose of control can be provided to the myoelectric hand based on the existing posture control logic.

**Figure 1:**
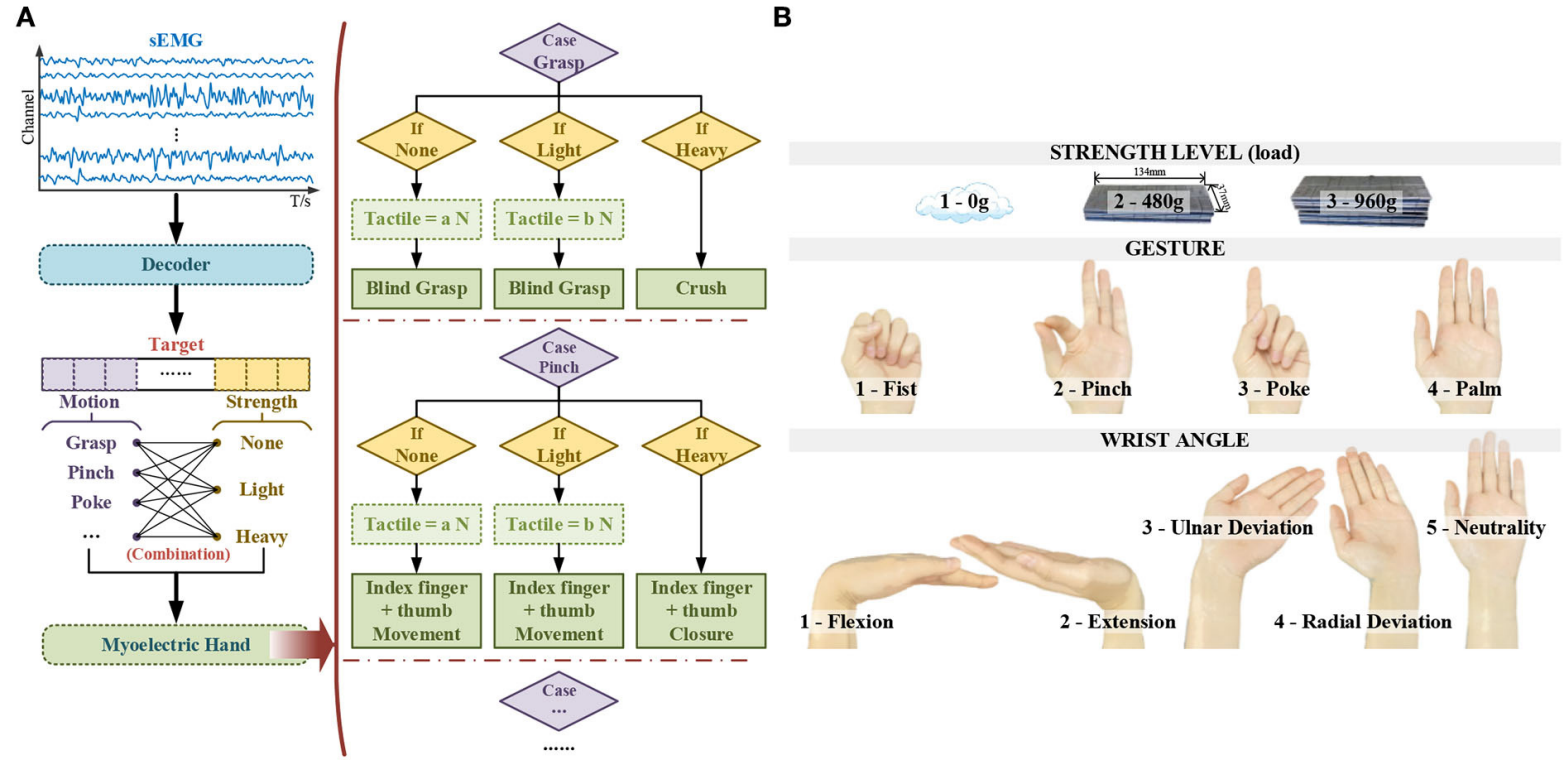
The selection basis of compound motions **(A)** the sEMG-based control strategy for the myoelectric hand **(B)** the illustration of compound motions.

Following the statement above, Figure 1A illustrates the sEMG-based control strategy for the myoelectric hand in this work.

#### Selection of compound motions

According to the control strategy, under different combinations of multi-targets decoded *via* sEMG, the myoelectric hand would adopt different logic to execute the desired movements.

As stated in Feix's report, there are 33 different grasp types in daily usage, including power and precision (Feix et al., 2016). By adopting this sEMG-based strategy above, for power blind grasping with all myoelectric fingers participating, the fist gesture (five-finger fist) was set as the enabling flag, which represents the grasp intention intuitively. Once enabled, according to the control strategy, the myoelectric hand would execute the blind grasp or the crush according to different strength levels. Besides the power grasp, the most representative pinch gesture (with index finger and thumb) was selected in the instruction set for precision grasp. Meanwhile, to fulfill the prior functionality, the poke (as one commonly used specific gesture with index finger stretch) and the palm (corresponding to the reset of myoelectric hand gesture) was also chosen as the sEMG-decoded gestures.

Since the gestures needed to be significantly decoded were reduced to only a few gestures, the core comes down to the decoding stability under various postures. Completing the hand task is inseparable from the flexible movement of the wrist. The angle of the wrist was taken into consideration to ensure the sEMG decoding stability. To ensure robust gesture decoding and further provide potential wrist control ability for the myoelectric prosthesis, discrete wrist angles were selected to form complex wrist-hand compound motions. The wrist has two degrees of freedom flexion/extension and radial/ulnar deviation. Thus, five discrete wrist angles were set, including flexion maximum, extension maximum, radial deviation maximum, ulnar deviation maximum, and neutrality position. As for the angle values, considering the individual differences and the feasibility when being applied to the myoelectric control, each maximum corresponds to the user's own limit.

Three strength levels were determined to increase the difference sufficient to switch the control purpose while providing adequate possibilities for subsequent development. Since the measurement of the strength level lacked calibration, as one initial work, different load levels were adopted to activate corresponding strength levels. Considering the experimental repeatability, the dimension of the adult's hand (Standardization, 1988) and counterweight, and the strength difference across genders, with the *Fe* adhesive weight, 0 g, 480 g, and 960 g, were selected. The underside of the weight was 134 mm in length and 37 mm in width, which fit the size of most adults' hands. Among three levels, 0 g represented the stably grip in blind grasping, 480 g (the approximated weight of a bottled drink) represented the grip with deformation, and 960 g represented the crush.

According to the basis above, by multiplying these four gestures, five wrist angles, and three strength levels, 60 modes decoded *via* sEMG were formed, as illustrated in Figure 1B.

### Materials

#### sEMG recording

**Figure 2:**
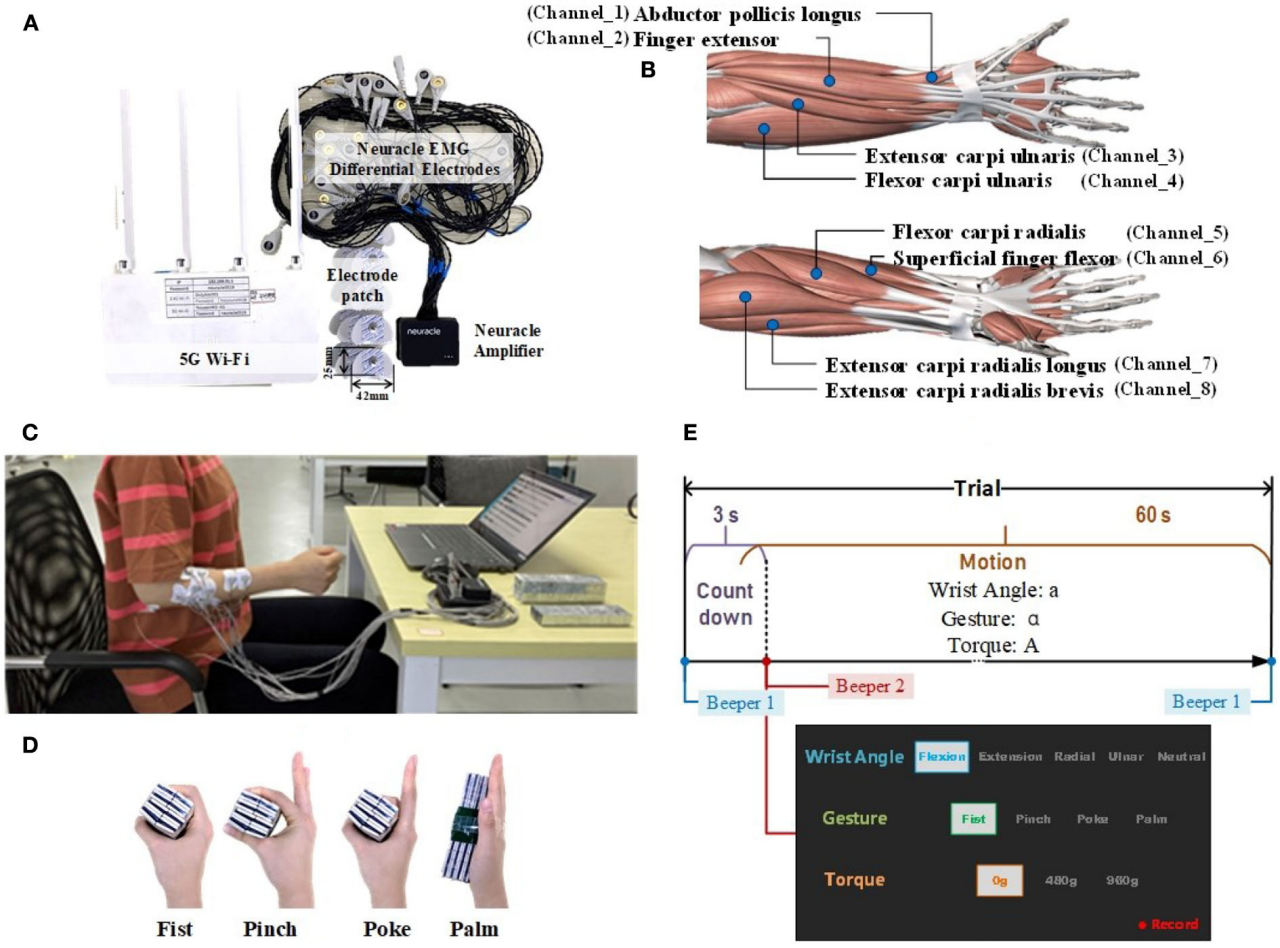
Experiment. **(A)** The Neuracle EMG acquisition system. **(B)** The electrode's placement. **(C)** The illustration of upper limb posture. **(D)** The way to load counterweight with different gestures. **(E)** The timing diagram of one session, along with the on-screen prompt example.

The commercial wireless portable EMG acquisition system (Neuracle Technology Co., Ltd., Changzhou, PRC) supporting up to 16 channels (each channel consisted of two surface differential electrodes) with a 1000 Hz sampling rate was adopted, as shown in Figure 2A. To decode the composite motion of fingers and wrist, sEMG electrodes were placed on the forearm. Eight large forearm muscles, which play a major role in grasping gestures and wrist movements, were selected, as illustrated in Figure 2B. Eight channels were targeted at these muscles with electrode patches (sized 42 mm in length and 25 mm in width), and the reference electrode was placed at the elbow. Before sticking the sEMG patches, alcohol was used to clean the skin.

#### Subjects

In total, 12 healthy subjects (aged 22–30 years, ten males and two females) participated in this study (Association, 2013). None of the subjects has a history of the upper extremity or other musculoskeletal complaints. Before starting, each subject was informed of the content, the purpose, and the detailed process of this experiment.

#### Experimental protocol

The experiment was conducted on the right arm. During the experiment, subjects sat with their elbows naturally hung down, and their forearms raised nearly horizontally. All the motions were completed with the palm kept vertically. The upper body posture is shown in Figure 2C.

In the experiment, each motion was recorded for one trial. Each trial began with a countdown for 3 s, followed by a motion mode hold for 60 s. Between every two trials, a 1-min break was arranged to avoid fatigue. A total of 60 trials were collected, corresponding to 60 modes (multiplied by four gestures, five wrist angles, and three strength levels). During the experiment, the loads were added to the hand. To stably add loads, tapes were attached to ensure the loads could be directly stuck to the palm. Figure 2D demonstrates how to load the counterweight with different gestures.

During the collection, subjects followed the on-screen prompt and the beeper to complete the specified motions. The timing diagram of each trial and the on-screen prompt example are illustrated in Figure 2E.

#### Dataset

For each subject, a total of 60 trials were collected. The first 20 trials were sEMG under-strength with 0 loads. The 21^st^-40^th^ trials were with a 480 g load, and the 41^st^-60^th^ trials were with a 960 g load. Each gesture was held for five trials in order at one strength level, with the wrist angle shifted in turn, according to the order in Figure 1B. The specific number ID of each compound motion is listed in Table 1. All data were preprocessed through detrending, the 2^nd^-order infinite impulse response notch filter at 50 Hz, and the 4^th^-order Butterworth bandpass filter at 20–250 Hz (Zhao et al., 2020).

**Table 1:** The specific number ID of each compound motion.

**Strength**	**0 load**					**480 g load**					**960 g load**				
**Wrist**	**Flexion**	**Extension**	**Ulnar**	**Radial**	**Neutrality**	**Flexion**	**Extension**	**Ulnar**	**Radial**	**Neutrality**	**Flexion**	**Extension**	**Ulnar**	**Radial**	**Neutrality**
Fist	1	2	3	4	5	21	22	23	24	25	41	42	43	44	45
Pinch	6	7	8	9	10	26	27	28	29	30	46	47	48	49	50
Poke	11	12	13	14	15	31	32	33	34	35	51	52	53	54	55
Palm	16	17	18	19	20	36	37	38	39	40	56	57	58	59	60

Considering the real-time performance of sEMG decoding, Lauer et al. (2000) stated that any delay greater than 200 ms would degrade the performance of one neuro-based task accomplishment. Taking the data acquisition and signal processing processes together, to ensure the system delay was less than 200 ms, data were sliced with a window length of 150 ms and 0 overlap. Thus, for each mode, there were 400 samples.

### Decoding methods comparison

**Figure 3:**
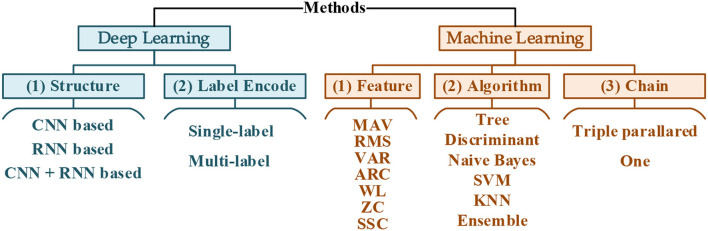
Summary of methods.

To study the decoding performance with these 60 compound motions, both deep learning and machine learning combined with different designs were evaluated, as summarized in Figure 3. The dataset from S1-S6 was adopted in the methods comparison. According to the chronological order, the first 90% of samples of each mode were selected as the training set (i.e., 1s−54s), and the rest were the validation set (i.e., 55s ~ the 60s). Within each dataset, the samples were shuffled.

#### Deep learning

##### Structure

The most well-known typical computations in deep learning were convolutional neural network (CNN), which originated from image decoding (Bengio and Lecun, 1997), and the recurrent neural network (RNN) from the natural language processing (Rumelhart et al., 1986). Developed from the RNN, the long short-term memory (LSTM) layers (Hochreiter and Schmidhuber, 1997) gained broader attention as its variant. Based on the CNN and the LSTM, lots of works achieved impressive results in sEMG decoding (Zhai et al., 2017; Hu et al., 2018; Rehman et al., 2018; Ameri et al., 2019). This work studied three structures (CNN-based, LSTM-based, and CNN+LSTM based) for their performance in 60 compound motion decoding.

**Figure 4:**
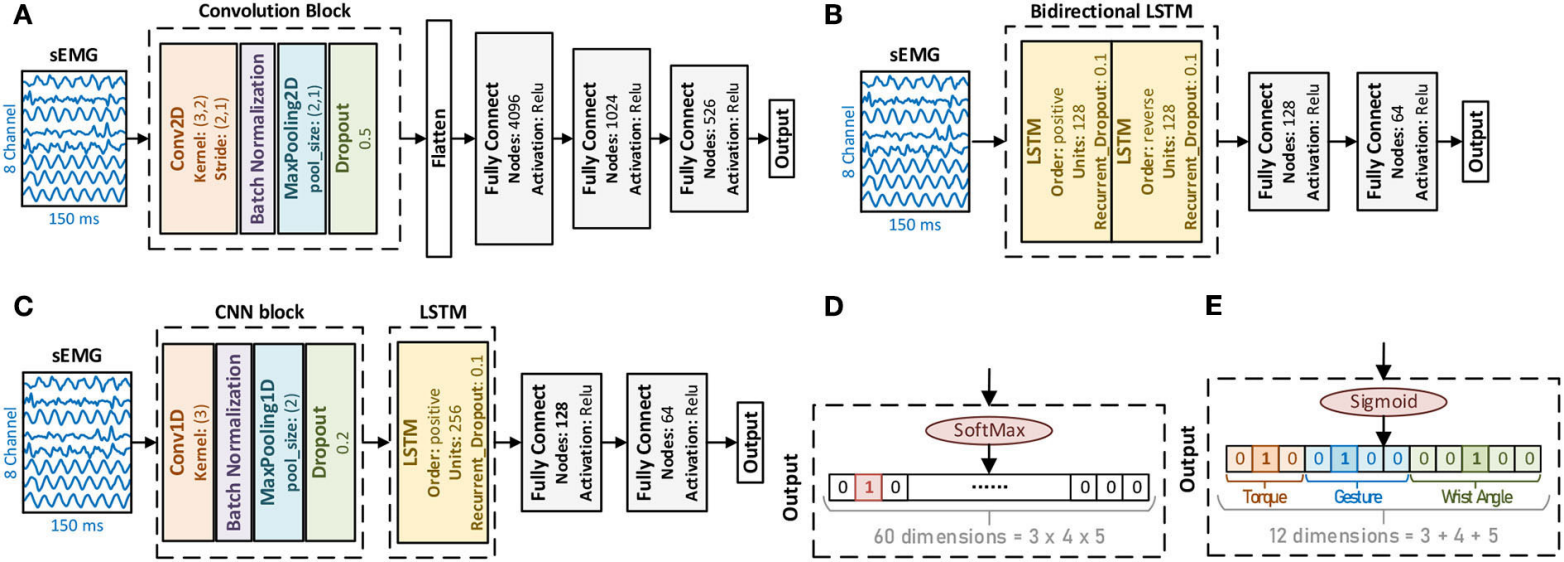
Deep learning methods have different structures and label encoding ways. **(A)** CNN-based structure. **(B)** LSTM-based structure. **(C)** CNN+LSTM-based structure. **(D)** One-hot label. **(E)** Multilabel.

**CNN-based:** In the CNN-based structure (Figure 4A), each convolution block consisted of a 2-dimensional convolution layer (Conv2D), a batch normalization layer (BN), a max pooling layer, and a dropout layer. For multi-convolution blocks, the number of filters descended, as the first block was 32 filters, the second was 16 filters, and the third was 8. For both 2 convolution blocks and 3 convolution blocks, the dense layer with 4096 nodes were deleted.

**LSTM-based:** Based on the RNN concept, the LSTM layer as a variant was adopted instead for better performance. Both the time positive and the time reverse order were considered the bidirectional LSTM, as illustrated in Figure 4B.

**CNN+LSTM-based:** This study adopted their combination to take advantage of CNN and LSTM. For the temporal sequence, the 1-dimensional convolution layer (Conv1D) along the temporal domain was taken first, followed by the LSTM, as in Figure 4C. For the convolution block, the same as the CNN-based structure, the first block comprised 32 filters.

##### Label encoding

Since these 60 compound motions consisted of gestures, wrist angles, strength levels, and two different ways of label encoding were adopted to compare their performance.

**One-hot label:** One-hot label treated compound motion decoding as a single-labeled multiclass problem. The corresponding output was 60 dimensions (Figure 4D). In accordance, the activation for the output layer was SoftMax.

**Multilabel:** By decomposing these compound motions into their corresponding modes in gesture, wrist angle, and strength, the problem could be transferred to the multilabel, multiclass classification. Thus, the output dimension was 12 (Figure 4E). For multilabel, the activation adopted was Sigmoid.

#### Machine learning

As mentioned in the dataset segment ahead, there were only 400 samples for each motion. The scale of the dataset was far from large. Thus, the traditional machine learning methods were also adopted in this work.

##### Feature

**Figure 5:**
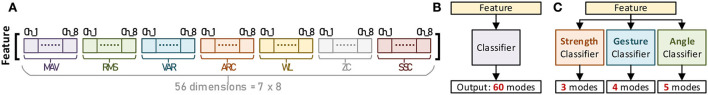
The feature vector and the classifier chain of machine learning methods. **(A)** Feature vector. **(B)** Integrated classifier. **(C)** Separated classifier.

Considering the short window length (150 ms), several commonly used features in the time domain were selected, such as mean absolute value (MAV), root means square (RMS), variance (VAR), 4^th^-autoregressive coefficient (ARC), wavelength (WL), zero crossings (ZC), and slope signal change (SSC) (Englehart and Hudgins, 2003; Zhao et al., 2020). Their mathematical definitions are listed in Table 2, where *x*_*i*_(*i* = 1, 2, ..., *N*) is the EMG time series, N equals 150 according to the window length, *a*_*k*_ is the autoregressive coefficient, and is the white noise. Different feature vectors can be formed through the permutations of these three features from eight sEMG channels. Combining these three features, a 56-dimension feature vector can be extracted in maximum, as shown in Figure 5A.

**Table 2:** The specific number ID of each compound motion.

**Feature**	**Mathematical definition**
MAV	${\text{T}}_{MAV}=\frac{1}{N}\overset{N}{\underset{i=1}{\sum }}|x_{i}|$
RMS	${\text{T}}_{RMS}=\sqrt{\frac{1}{N}\overset{N}{\underset{i=1}{\sum }}{x_{i}}^{2}}$
VAR	${\text{T}}_{VAR}=\frac{1}{N-1}\overset{N}{\underset{i=1}{\sum }}{\left(x_{i}-\bar{x}\right)}^{2}$
4^th^-ARC	$x_{i}=-\overset{4}{\underset{k=1}{\sum }}a_{k}x_{i-k}+\omega_{i}$
WL	${\text{T}}_{wl}=\overset{N-1}{\underset{i=1}{\sum }}|x_{i+1}-x_{i}|$
ZC	${\text{T}}_{zc}=\overset{N-1}{\underset{i=1}{\sum }}\text{sgn}\left(-x_{i}x_{i+1}\right)$
SSC	(*x*_*i*_ − *x*_*i* − 1_) × (*x*_*i*_ − *x*_*i* + 1_) ≥ ω, where ω = 0.05*std*

##### Algorithm

Various types of classifiers were adopted to evaluate the decoding performance, including tree, discriminant, support vector machine (SVM), K-nearest neighbor (KNN), and some ensemble methods. The details of these 24 algorithms are listed in Table 3, where *G*(*x*_*i*_, *x*_*j*_).denotes elements in the gram matrix, *x*_*i*_, *x*_*j*_ denote the observations, and γ is the width of the Gaussian kernel.

**Table 3:** The specific number ID of each compound motion.

**Algorithm**	**Subdivided**	**Attribute**		**Notes**	
Decision trees	Fine	Maximum leaf	100	Split criterion	Gini Index
	Medium	Maximum leaf	20		
	Coarse	Maximum leaf	4		
Discriminant	Linear	\		\	
	Quadratic	\		\	
Naïve bayes	Gaussian	Distribution	Normal		
	Kernel	Type	Gaussian	Width	Automatic
SVM	Linear	Kernel	$G\left(x_{i},x_{j}\right)={x_{i}}^{\prime }x_{j}$		
	Quadratic		$G\left(x_{i},x_{j}\right)={\left(1+{x_{i}}^{\prime }x_{j}\right)}^{2}$		
	Cubic		$G\left(x_{i},x_{j}\right)={\left(1+{x_{i}}^{\prime }x_{j}\right)}^{3}$		
	Fine gaussian		$G\left(x_{i},x_{j}\right)=\exp\left(-{\left||x_{i}-x_{j}|\right|}^{2}/\gamma \right)$	Scale γ	0.56
	Medium gaussian		$G\left(x_{i},x_{j}\right)=\exp\left(-{\left||x_{i}-x_{j}|\right|}^{2}/\gamma \right)$	Scaleγ	2.2
	Coarse gaussian		$G\left(x_{i},x_{j}\right)=\exp\left(-{\left||x_{i}-x_{j}|\right|}^{2}/\gamma \right)$	Scaleγ	8.9
KNN	Fine	Distance	$d_{st}^{2}=\left(x_{i}-x_{j}\right){\left(x_{i}-x_{j}\right)}^{\prime }$	Neighbors	1
	Medium		$d_{st}^{2}=\left(x_{i}-x_{j}\right){\left(x_{i}-x_{j}\right)}^{\prime }$	Neighbors	10
	Coarse		$d_{st}^{2}=\left(x_{i}-x_{j}\right){\left(x_{i}-x_{j}\right)}^{\prime }$	Neighbors	100
	Cosine		$d_{st}=(1-x_{i}x_{j}{}^{\prime }/\sqrt{\left(x_{i}x\prime_{i})(x_{j}x\prime_{j}\right)}\;)$	Neighbors	10
	Cubic		$d_{st}=\sqrt[{3}]{\overset{n}{\underset{u=1}{\sum }}{\left|x_{iu}-x_{ju}\right|}^{3}}$	Neighbors	10
	Weighted		$d_{st}^{2}=\left(x_{i}-x_{j}\right)V^{-1}{\left(x_{i}-x_{j}\right)}^{\prime }$	Neighbors	10
Ensemble	Boosted trees	\		\	
	Bagged trees	\		\	
	Subspace discriminant	\		\	
	Subspace KNN	\		\	
	RUSBoosted trees	\		\	

##### Chain

Similar to the label encoding way in deep learning, the structure of machine learning classifiers can also be designed in one integrated or three separated ways (Figures 5B,C). There was a single classifier decoding 60 categories for the integrated structure. The separate, triple-parallel structure was set, and each took charge of strength, wrist angle, or gesture separately.

### Compound motion decoding

Throughout the methods above, the best was selected under the comparison with average accuracy, prediction speed, training speed, and so on. Then, with the best decoding method, datasets from S1-S12 were all adopted to provide a detailed decoding performance analysis among all these 60 compound motions. Similarly, according to the chronological order, the first samples of each mode were selected as a training set, and the rest were test-set. Within each dataset, the samples were shuffled.

To focus more on the decoding performance among all 60 compound motions, the analysis included (1) the change of test accuracy with the decrease of the training set; (2) the test accuracies of each motion and their confusion matrix; (3) and the test accuracy for separate motions of strength levels, gestures, and wrist angles.

## Results

### Decoding methods comparison

#### Deep learning

Through comparisons, considering the accuracy, stability, overfitting, and training time, the CNN+LSTM-based structure with one-hot label achieved the best performance at 94.61 ± 3.20% for training and 94.20 ± 4.06% for validation. The details are given below.

##### Structure

**Figure 6:**
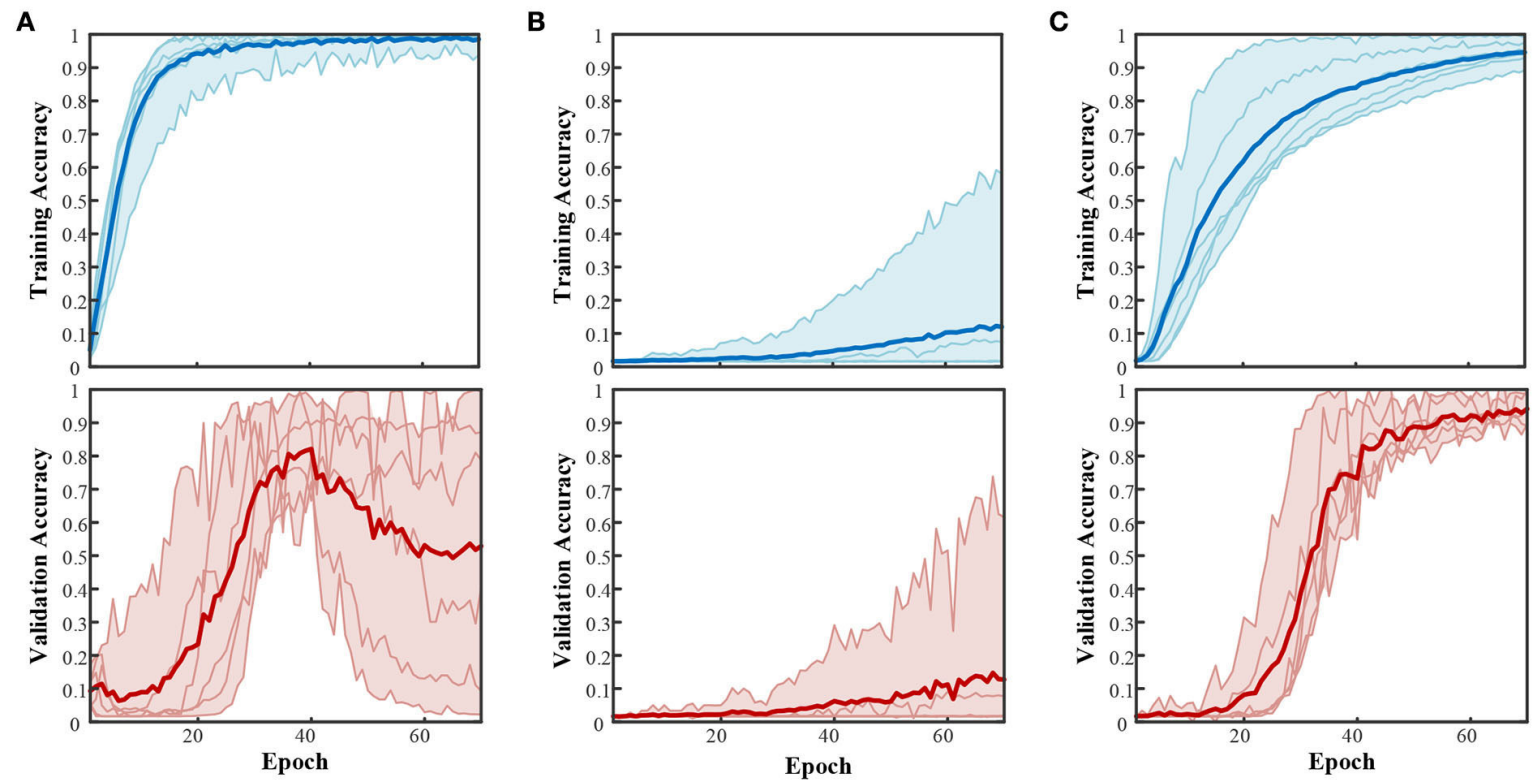
Accuracy curves during training and validation with three structures. **(A)** CNN-based structure with a one-hot label. **(B)** LSTM-based structure with a one-hot label. **(C)** CNN+LSTM-based structure with a one-hot label.

The TensorFlow (Abadi et al., 2016) 2.0 framework was adopted as the supporting backend for deep learning realization. The optimizer and batch size remained the same for CNN-based, LSTM-based, and CNN+LSTM-based structures (Table 4). At first, a one-hot label connected with SoftMax activation was adopted. The training and validation curves are illustrated in Figure 6.

**Table 4:** Compile setting in structure comparison.

**Type**	**Method**	**Parameter**	**Value**
Optimizer	RMSprop	Learning rate	0.0008
		Clip value	1.0
		Decay	1e-8
Training	Batch	Batch size	1,024
	Epoch		70

The subject-average accuracy curve in Figure 6 indicates that the CNN+LSTM-based structure outstands these three structures. The final subject-averaged training and validation accuracies for CNN+LSTM-based are 94.61 ± 3.20% and 94.20 ± 4.06%. Especially for subject S4, the validation accuracy approaches 99.77% for 60 modes with CNN+LSTM. Structure (a) with only one convolution block showed an ideal training curve but poor validation. An extensive validation decay in the post-training period emerged from S3, S5, and S6 (9.36, 2.19, and 40.36%); for S1, S2, and S4, the validation accuracy ended at 78.72, 87.19, and 99.47%. Such huge individual differences indicate the instability of structure (a) in this compound motion decoding. For bidirectional LSTM-based structure (b), the highest validation accuracy was 61.38% by S4, while the rest of the subjects remained below 10%. Under the same configuration environment, with Win 10, i5-6500 (3.20 GHz), and GTX 960, the average time cost for training 70 epochs was 278.91s for CNN-based, 1,491.74s for LSTM-based, and 528.94s for CNN+LSTM based.

**Figure 7:**
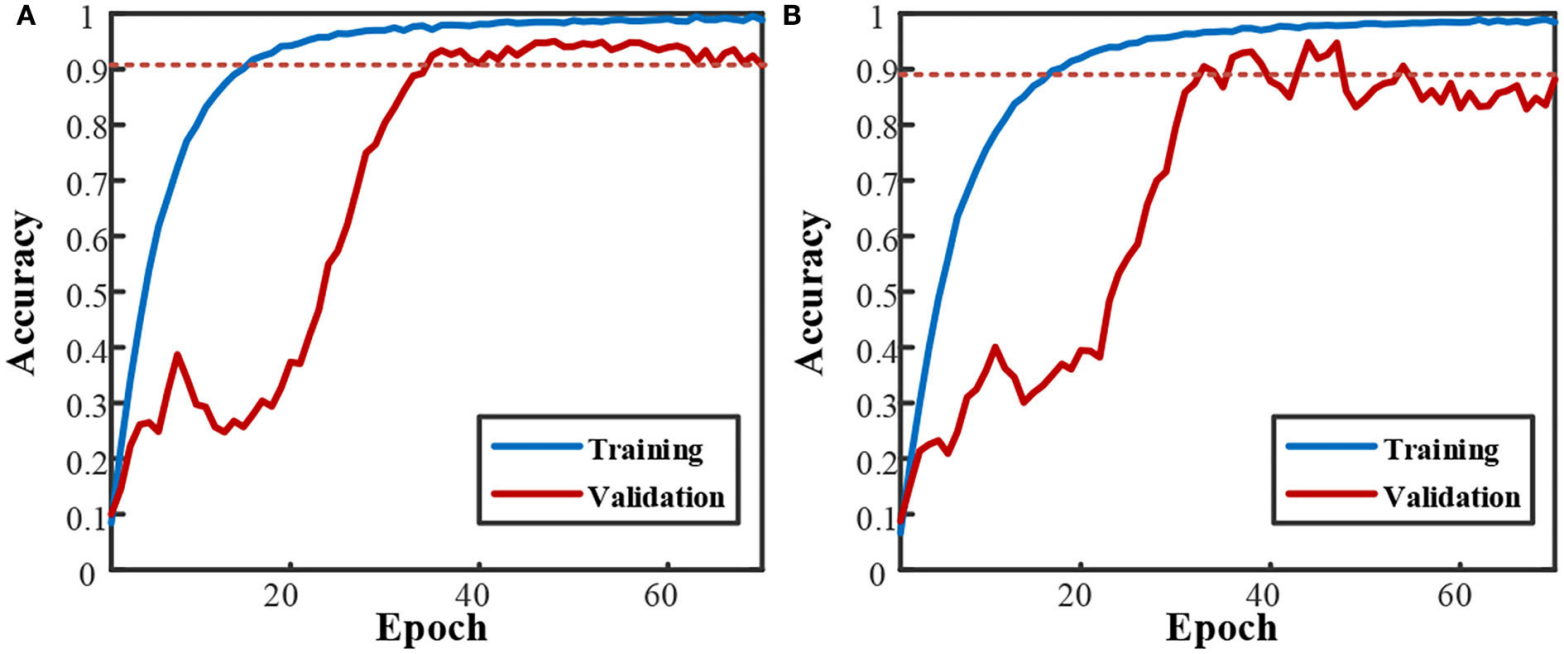
Subject-averaged training and validation performance of CNN based networks with increased convolution block. **(A)** 2 CNN blocks. **(B)** 3 CNN blocks.

For the CNN-based network, with the increase of convolution blocks and the deletion of the dense layer (4,096 nodes), the final decay in Figure 6 has been greatly relieved, as shown in Figure 7. Compared with only one convolution block in Figure 6, the validation performances are largely improved, ending at nearly 90%. However, more blocks led to higher overfitting, with the gaps between training and validation being 8% for two blocks and 10% for three blocks. The early decline in the validation curve indicated that great overfitting occurred in the early stage due to insufficient training. The validation accuracy is improved with the adjustment of convolution blocks but is within the CNN+LSTM. The average training time cost for two blocks was 76.90 s and 56.54 s for three blocks. The training was accelerated by reducing trained parameters as the block increased.

##### Label encoding

**Figure 8:**
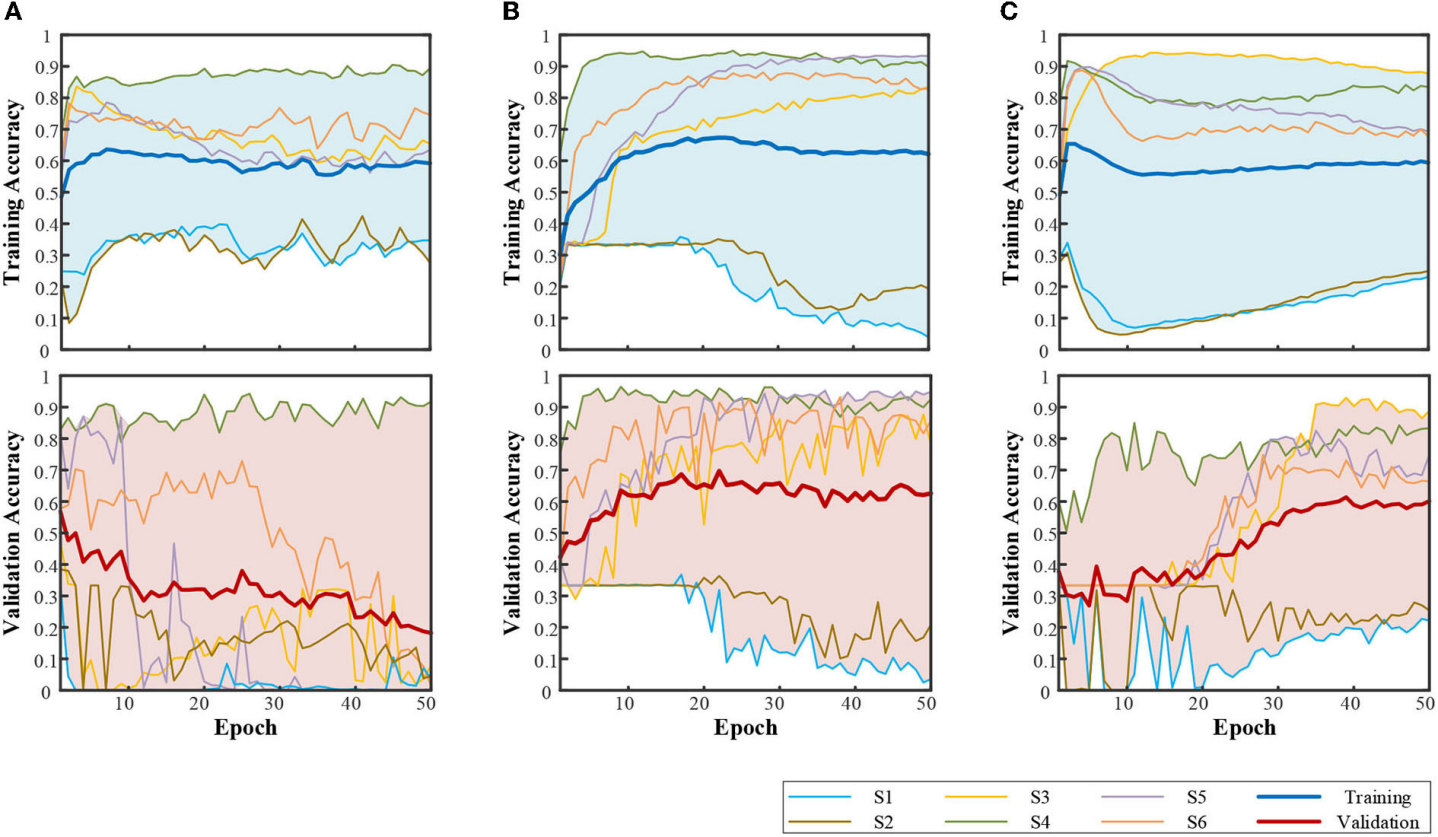
Performance with multilabel. **(A)** CNN-based structure. **(B)** LSTM-based structure. **(C)** CNN+LSTM-based structure.

The influence of multilabel is shown in Figure 8. The performance of the one-hot label is already shown in Figure 6. The compilation was kept the same as in Table 4. Compared with the one-hot label, the filling part in Figure 8 shows a larger individual difference, led by the multilabel. For bidirectional LSTM, the performance of S3–S6 was significantly improved with multilabel, while there was no help for S1 and S2. It illustrates that multilabel classification of compound motion resulted in greater instability.

#### Machine learning

For this relatively small sample multiclass sEMG classification, combined with feature engineering, several machine learning classifiers show great applicability and excellent performance. Through comparison, the quadratic SVM for 60 modes once achieved the best (*p* = 0.04 < 0.05 with *T*-Test). The details are as given below.

##### Algorithm

The averaged validation accuracy of 60 modes from various machine learning classifiers is listed in Table 5, along with their training times and prediction speeds. In Table 5, among all 24 classifiers, the average accuracies of 16 classifiers exceed 90%, and ten classifiers exceed 95%. Such generally high performance demonstrates the applicability of traditional feature extraction and machine learning methods in the small sample multiclass sEMG decoding. Among all classifiers, the SVM with a quadratic kernel achieved the highest subject-averaged accuracy at 98.23 ± 1.51%. Its 1.5% standard deviation indicates a small individual difference among subjects and stable overall performance. In terms of time spent, the average time cost for quadratic SVM training is 443.8 s without acceleration.

**Table 5:** Comparison of machine learning classifiers.

**Model type**		Accuracy*(%)								Training time*(sec)	Prediction Speed*(obs/sec)	Accuracy*(%)		
		**S1**	**S2**	**S3**	**S4**	**S5**	**S6**	**AVE**	**Std Dev**			**Strength**	**Gesture**	**Angle**
Tree	Fine	63.9	63.9	63.4	99.9	63.7	99.0	75.63	16.84	\	\	96.50	93.05	85.38
	Medium	27.4	22.9	30.0	35.0	29.7	35.0	30.00	4.23	\	\	94.20	78.30	66.87
	Coarse	6.6	6.5	8.2	8.3	7.0	8.3	7.48	0.80	\	\	88.72	57.68	47.73
Linear discriminant	90.7	90.3	91.9	99.5	96.7	99.7	94.80	3.98	11.3	228.3	92.98	77.95	72.02
Quadratic discriminant	94.2	94.5	95.2	\	99.3	99.9	\	\	\	\	92.57	89.75	87.73
Naive bayes	Gaussian	90.2	91.0	87.1	99.9	96.9	99.6	94.12	4.93	\	\	83.45	56.32	47.50
	Kernel	92.1	93.8	92.3	99.6	97.9	99.6	95.88	3.25	619.3	429.3	91.65	79.93	\
SVM	Linear	96.0	96.1	96.4	100.0	98.8	99.8	97.85	1.73	467.0	696.7	94.28	87.72	81.70
	Quadratic	96.5	96.5	97.3	100.0	99.2	99.9	98.23	1.51	443.8	586.7	98.35	98.57	97.35
	Cubic	96.2	96.2	97.1	100.0	99.1	99.9	98.08	1.64	452.0	485.0	\	\	\
	Fine Gaussian	\	\	\	95.6	\	\	\	\	\	\	98.62	98.13	97.27
	Medium Gaussian	95.4	95.8	96.0	99.9	98.9	99.9	97.65	1.95	538.8	421.7	98.48	98.17	96.00
	Coarse Gaussian	93.5	93.9	94.0	100.0	97.9	99.7	96.50	2.78	539.2	418.3	94.18	84.52	74.28
KNN	Fine	91.0	90.2	94.5	100.0	97.4	99.7	95.47	3.89	153.4	730.0	98.63	98.33	97.30
	Medium	91.9	91.0	94.5	99.9	97.2	99.7	95.70	3.51	158.8	768.3	98.68	98.38	97.38
	Coarse	88.0	87.2	90.5	99.4	94.1	99.3	93.08	4.94	\	\	97.70	97.05	95.60
	Cosine	91.0	89.6	93.2	100.0	96.8	99.6	95.03	4.04	\	\	98.52	98.02	97.03
	Cubic	91.0	89.9	\	99.9	\	\	\	\	\	\	\	\	\
	Weighted	92.4	91.7	94.8	100.0	97.5	99.7	96.02	3.29	196.7	935.0	98.80	98.52	97.65
Ensemble	Boosted Trees	65.2	62.4	59.0	99.9	73.8	97.3	76.27	16.43	\	\	73.52	89.52	77.18
	Bagged Trees	94.7	94.8	95.6	100.0	98.3	99.7	97.18	2.23	225.7	661.7	98.98	98.55	97.57
	Subspace Discriminant	88.0	87.8	90.2	99.4	95.1	99.6	93.35	4.97	\	\	91.62	71.85	65.47
	Subspace KNN	89.3	88.3	93.4	99.9	97.4	99.5	94.63	4.64	\	\	\	97.70	\
	RUSBoosted Trees	26.2	21.3	30.0	35.0	29.7	35.0	29.53	4.81	\	\	71.97	78.32	66.87

*Evaluated on the Win10, AMD R7 (integrated graphics). The ‘ín accuracy indicated that the training time for this classifier exceeded 1,000 s. The ‘ín training time and prediction speed indicated that the performance of this classifier did not meet expectations, so no analysis was done.

##### Chain

The subject-averaged validation accuracies using the triple-parallel classifier chain for decoding the gestures, the wrist angles, and the strength levels are presented in Table 6. The highest accuracies were achieved by subspace discriminant for three strength levels (98.98 ± 1.51%); quadratic SVM for four gestures (98.57 ± 1.15%); and weighted KNN for five wrist angles (97.65 ± 1.72%). By combining these three classifiers to form the triple-paralleled chain, we can see that the theoretical decoding accuracy was the product of three accuracies, which equals 92.93, 90.79, 92.47, 100.00, 96.05, and 99.60% for S1~S6. The average subject accuracy for the classifier chain was 95.30 ± 3.54%. The classifier chain did not show superiority compared with the one integrated classifier.

**Table 6:** Averaged validation accuracies for triple-paralleled classifier chain.

**Model type**		**Accuracy (%)**		
		**Strength level**	**Gesture**	**Wrist angle**
Tree	Fine	96.50	93.05	85.38
	Medium	94.20	78.30	66.87
	Coarse	88.72	57.68	47.73
Linear discriminant		92.98	77.95	72.02
Quadratic discriminant		92.57	89.75	87.73
Naive bayes	Gaussian	83.45	56.32	47.50
	Kernel	91.65	79.93	\
SVM	Linear	94.28	87.72	81.70
	Quadratic	98.35	98.57	97.35
	Cubic	\	\	\
	Fine Gaussian	98.62	98.13	97.27
	Medium Gaussian	98.48	98.17	96.00
	Coarse Gaussian	94.18	84.52	74.28
KNN	Fine	98.63	98.33	97.30
	Medium	98.68	98.38	97.38
	Coarse	97.70	97.05	95.60
	Cosine	98.52	98.02	97.03
	Cubic	\	\	\
	Weighted	98.80	98.52	97.65
Ensemble	Boosted Trees	73.52	89.52	77.18
	Bagged Trees	98.98	98.55	97.57
	Subspace Discriminant	91.62	71.85	65.47
	Subspace KNN	\	97.70	\
	RUSBoosted Trees	71.97	78.32	66.87

##### Feature

**Figure 9:**
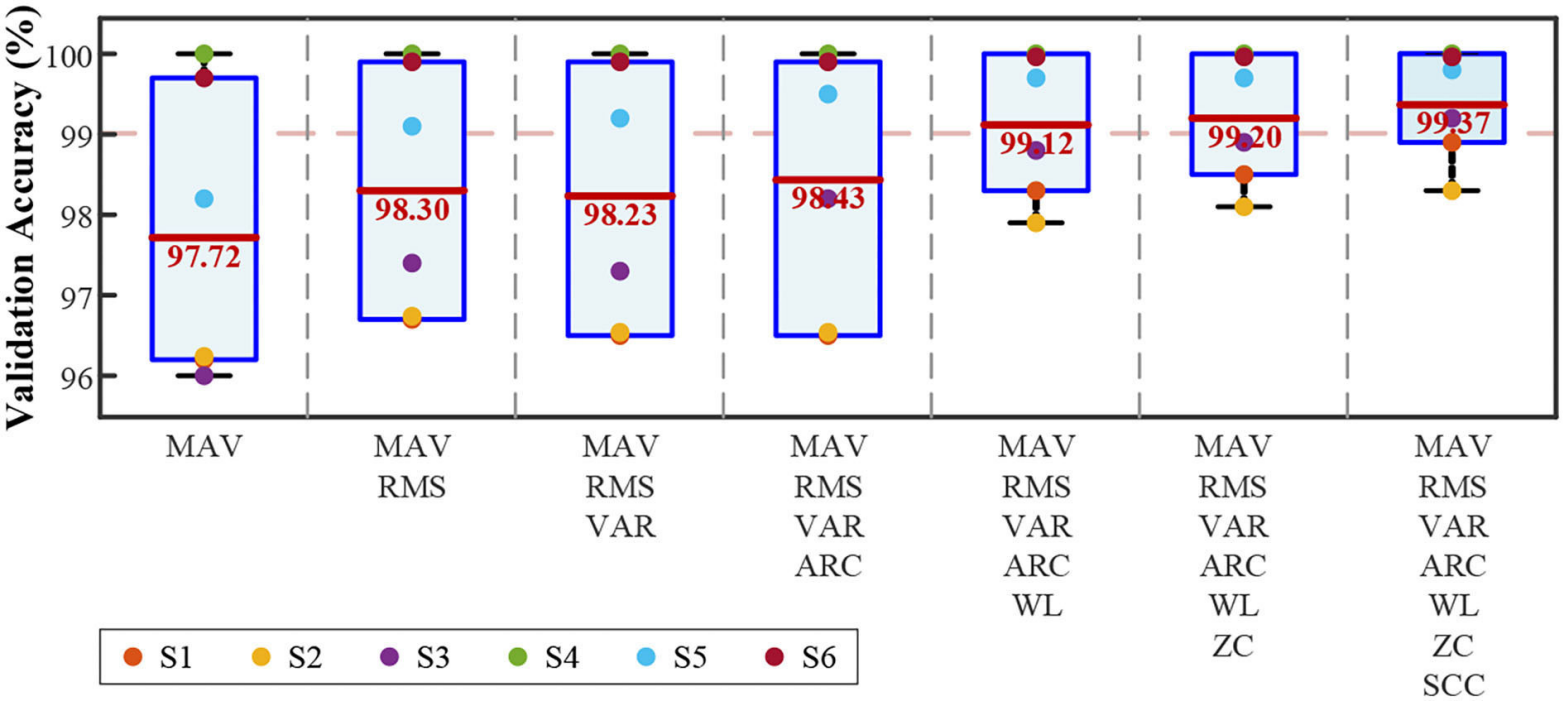
Validation accuracies of quadratic SVM with an increased number of features.

In total, seven kinds of temporal features were adopted. Figure 9 illustrates the change in validation accuracy as the number of features increases. As the number of features increases, the average validation accuracy and the standard deviation decrease from 1.68 to 0.63%. The performance remained above 99.00% in the range of 5–7 features. Among them, the combination of all seven features was the best.

### Compound motion decoding

**Figure 10:**
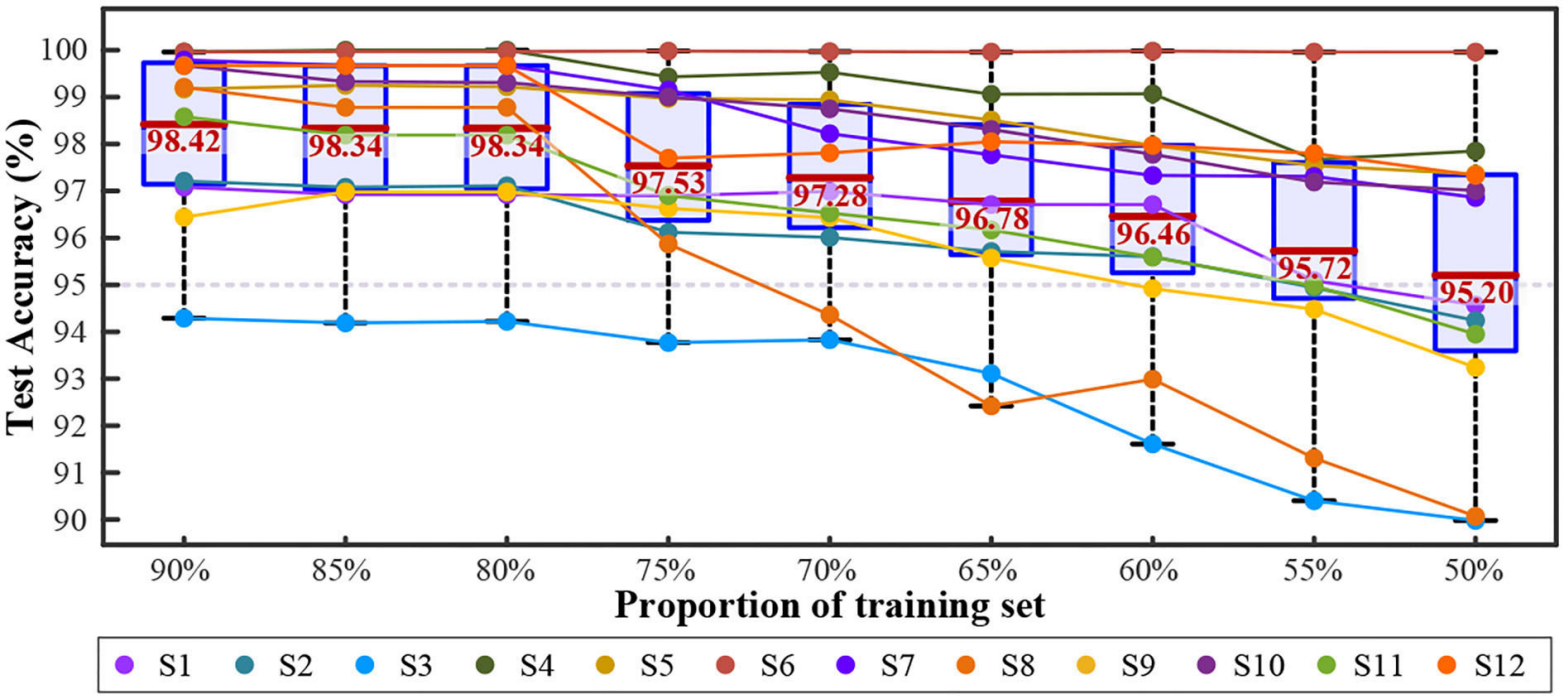
The change of validation accuracies with quadratic SVM combining seven features.

In the overall comparison, the SVM with a quadratic kernel performed the best after 443.8 s of training. Feature engineering was the combination of all seven features. Figure 10 depicts the variation in test accuracy resulting from altering the proportion of the training set to the testing set for such a decoding method. In each mode, the latter data were used for testing.

**Figure 11:**
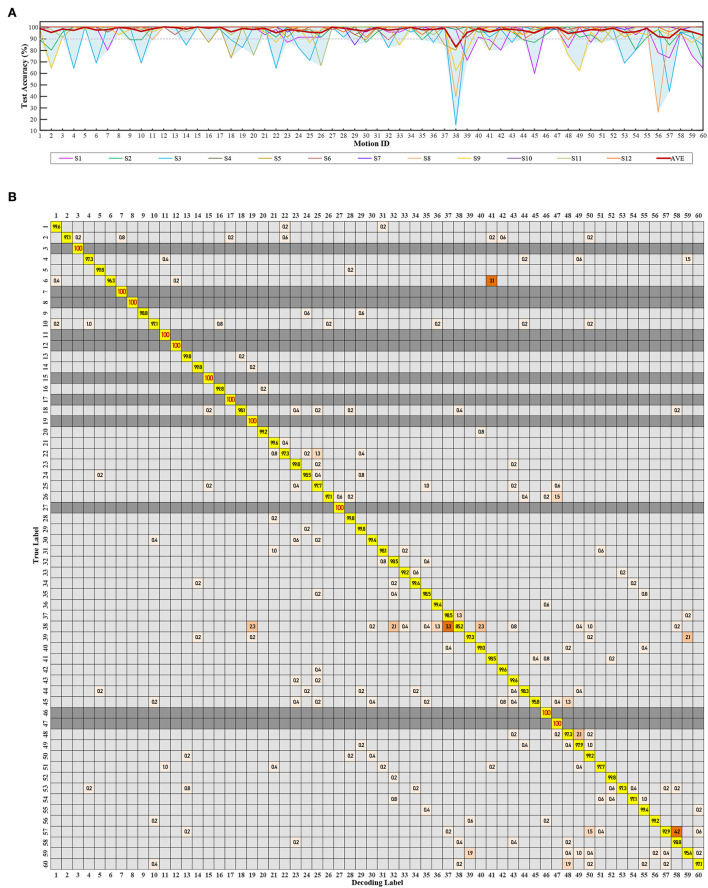
Testing performance of 60 compound motions with quadratic SVM combining seven features. The label 1~60 match Table 1. **(A)** The validation and test accuracy. **(B)** Subject average confusion matrix of 60 compound motions.

As the training proportion decreased from 90 to 50%, the average test accuracy remained higher than 95%, which showed great generalization. However, the standard deviation increased from 1.71 to 2.96%, and the individual differences became prominent. Six of the twelve subjects kept their test accuracy higher than 95% in all the processes, while S3 and S8 gradually dropped to approach 90%. S3 performed the worst (from 94.29 to 89.98%). S8 decreased the most (from 99.21 to 90.07%). S6 achieved the most stable performance (with an average accuracy of 99.97 ± 0.01% from 90% training to 50%). Figure 11A shows the test accuracy of each mode with a 90% training set.

The average test accuracy with a 90% training set was 98.42 ± 1.71%. Among all subjects, S4 and S6 achieved the best at 99.96 ± 0.32%. Four of these twelve subjects maintained their accuracies for 60 modes to be all greater than 95%, and most were equal to 100%. Figure 11B is the confusion matrix.

Within 60 modes in Figure 11B, eleven modes achieved 100% test accuracy for all twelve subjects. Thirty-one modes are higher than 99%. Fifty-eight modes are higher than 95%. Motion 38 [palm, ulnar deviation, 480g], and motion 57 [palm, extension, 960g] performed lower than 95%, with motion 38 the worst at 85.2%. Table 7 lists the test accuracy for separate motions, which achieved 99.35 ± 0.67% for three classes of strength-level decoding, 99.34 ± 0.88% for four classes of gesture decoding, and 99.04 ± 1.16% for five classes of wrist-angle decoding. Meanwhile, since the addition of wrist angles ensures decoding stability in various postures, the average test accuracy of “strength + gesture” is 98.95 ± 1.11%.

**Table 7:** The test accuracy for separate motions of strength levels, gestures, and wrist angles with quadratic SVM combining seven features.

**Subject**	**Test accuracy (%)**			
	**Strength**	**Gesture**	**Angle**	**Strength+Gesture**
S1	98.54	98.71	98.08	97.63
S2	98.25	99.04	99.13	97.54
S3	98.71	97.00	95.83	96.92
S4	100.00	100.00	99.96	100.00
S5	100.00	99.92	99.17	99.92
S6	100.00	100.00	99.96	100.00
S7	99.96	99.79	99.96	99.79
S8	99.54	99.96	99.25	99.50
S9	98.58	98.38	98.13	97.79
S10	99.96	99.75	99.96	99.71
S11	98.79	99.58	99.33	98.75
S12	99.83	99.96	99.79	99.83
AVE	99.347	99.340	99.045	98.948
Std	0.674	0.880	1.159	1.109

## Discussion

This study stated the need for compound motion decoding in myoelectric control and further investigated and realized the classification of 60 compound motions with 150 ms sEMG collected from eight forearm muscles. Different methods of deep learning and machine learning were adopted to assess their capability. In deep learning, three structures and two ways of label encoding were analyzed. Among them, the CNN+LSTM with a one-hot label performed the best. In machine learning, 24 classifiers, different combination of features, and classifier chain were tested. The quadratic SVM combined with seven features showed the highest validation accuracy and the smallest variance. Compared with deep learning, classifiers from machine learning showed more stability and robustness. Overall, the quadratic SVM exceeded the CNN + LSTM with higher validation accuracy, lower training time, and less variance. This result demonstrated the ability of traditional machine learning on relatively small sample sEMG multi-classification problems.

### The significance and the performance

Considering the ultimate goal to be a more flexible control of the myoelectric hand by incorporating the blind grasp, this study proposes the need for sEMG-based compound motion decoding, paying particular attention to the need for control purpose switching (such as grip vs. crush), the differentiation between power grasp and precision grasp, and the manipulation of the wrist joint. With the 60 compound motions in this work (as the product of four gestures, five wrist angles, and three strength levels), we shall not only guarantee the flexibility of control ability but also guarantee the stability of sEMG-based decoding under a variant upper limb posture.

In comparing deep learning methods and machine learning classifiers, facing the same ultimate goal as flexible control, besides the accuracy, the training time, the prediction speed, and the dependence on computing hardware, all matter. Combined with manual feature engineering, most classifiers in Table 5 showed appreciating results with small variance among subjects. While among the three structures in the deep learning method, only CNN + LSTM steadily converged (Figure 6). This result indicates that the information contained in the short-windowed original sEMG is sparse and chaotic. The capability of simply designed CNN or LSTM in auto feature extraction is limited in this multi-classification with only small samples. This affirms the value of traditional manual feature engineering in small-sample multiclass sEMG decoding.

With the help of this manual feature engineering, the training process of machine learning classifiers was speedy. For most classifiers, without the acceleration of GPU, the training process can still be kept for approximately 5 mins. Conversely, the shorter time for deep learning is consistent with the longer time in machine learning.

As for stability, since the SVM has a high generalization, although the accuracy decreases with the training proportion, the overall performance was still acceptable. For the balance among 60 modes, considering the individual differences, the standard deviation varied from 0.32% (S4, S6) to 14.74% (S3). Two subjects showed excellent decoding performance, with the standard variance close to 0.00%.

In conclusion, with the addition of feature extraction, the machine learning approaches in this very small sample multiclass sEMG compound motion decoding stand out for their excellent accuracy, fast training procedure, low computation cost, and stability.

### The limitation and future work

#### Experiment protocol

In the materials, limited by the size of the electrode patches adopted in this study, targeted placement was adopted instead of equally spaced. However, several studies have reported that the equally spaced placement achieved better performance for the machine learning method. In future research, tinier patches will be used to compare the performance under different electrode placements. Meanwhile, it has been noticed that the motions executed in sequence might increase the inter-class difference and decrease the intra-class difference. This may lead to a seemingly appropriate decoding performance. In future work, we are considering further reducing the data collection work of each motion and improving the data collection scheme to be decentralized and disordered.

#### Decoding performance

In the subsequent research work, feature engineering with the quadratic SVM resulted in regretful test accuracies for the contralateral decoding and cross-subjects. This demonstrated that manual feature engineering has distinct personal characteristics and that transferring the trained network to other people or extra objects is difficult. However, for deep learning, several papers reported the transfer learning ability in cross-subject sEMG decoding. In 2021, Chen constructed a CNN-based general gesture EMG feature extraction network of 30 hand gestures, then transferred it into the decoding of extra gestures, which improved the recognition accuracy by 10 and 38% (Chen et al., 2021). Jiang proposed a correlation-based data weighting method that achieved a low root mean square error in cross-subject evaluation with significant performance improvement (Hautier et al., 2000). Based on CNN, Yu proposed a transfer learning strategy for instantaneous gesture recognition that improved the average accuracy of new subjects and new gesture recognition by 18.7 and 8.74% (Yu et al., 2021). In 2017, Cote-Allard used the CNN-based transfer learning techniques to leverage inter-user data from the first dataset and alleviate the data generation burden imposed on a single individual (Cote-Allard et al., 2017). The above research makes us believe that, with the help of transfer learning, deep learning is more suitable for cross-subject and cross-object research. However, manual feature engineering and machine learning still have a place in subject-specific decoding with small samples and large categories. Further, the paper lacks online validation on amputees as an initial work. When verifying the feasibility of compound motion decoding under a 150 ms window length, some degradation of decoding accuracy may occur when applied to online decoding. Meanwhile, the study on decoding methods, the study of blind-grasp, and the research on improving stability and reducing noise interference are equally important for amputees' successful online task operation.

#### Improvements in compounded motions

As for the selection of compound motions, considering the repeatability and controllability during the experiment, loads with counterweights are used to activate the strength levels. However, such specified strength levels can hardly be reproduced in the realistic online control of the myoelectric hand for the disabled. Since the separability of three levels of strengths in 60 compound motions has been demonstrated, future research will emphasize the practicality by replacing strength levels with three different loads (stuck to the hand) with one's maximum strength, medium strength, and weak strength, thereby realizing the switch of control purposes during the online control of the myoelectric hand. Meanwhile, with the more complex design of the myoelectric hand's control purpose, the number of strength levels would be increased according to the demand of the control purposes. Next, on the premise of stability, more gestures would be included to enlarge the instruction sets. The four gestures now selected in this work were all functional gestures, and none of them was sign language. In the following research, according to the proposed control logic, besides the functional gestures, more sign languages are planned to be added to enrich the communicational usage of the myoelectric hand. Moreover, based on this work, transfer learning is planned to be studied next for the adaptation of more users and more complex and personalized decoding sets.

## Conclusion

Considering the control purpose switching (such as grip vs. crush), the distinction between power grasp and precision grasp, and the manipulation of the wrist joint in the control of the myoelectric hand, this work puts forward the need for compound motion decoding. With 150 ms sEMG from eight muscles, decoding 60 upper limb compound motions achieved an average accuracy of 98.42 ± 1.71%. These 60 motions were the product of four gestures, five wrist angles, and three strength levels. Among all 60 motions, 48 showed a test accuracy greater than 95%, and one part was equal to 100%. In comparing decoding performance, several deep learning methods and machine learning classifiers were adopted, with the contrast among structures, label encoding ways, and algorithms. The feature engineering (MAV+RMS+VAR+ARC+WL+ZC+SSC) combined with the SVM (quadratic kernel) stood out for its high accuracy, short training process, less computation cost, and well stability (*p* < 0.05). The comparison results highlighted the value of manual feature engineering and machine learning classifiers in relatively small sample multiclass sEMG decoding. As a prerequisite work for myoelectric control, this study provides a flexible solution for the subsequent involvement of blind grasping and control purposes, aiming to provide a more stable, diversified, and convenient operation for the myoelectric hand.

## Data availability statement

The raw data supporting the conclusions of this article will be made available by the authors, without undue reservation.

## Ethics statement

The experiment in this study was approved by the Ethics Committee (Institutional Review Board) of Xi'an Jiaotong University (No. 20211452). The patients/participants provided their written informed consent to participate in this study.

## Author contributions

XZ supervised this work and revised the manuscript. ZL proposed, did the research, and wrote the manuscript. CF edited the manuscript and financially supported the publication. YW, TZ, and HL organized and carried out the experiments. QT revised the manuscript. All authors contributed to the article and approved the submitted version.

## Funding

This work was supported in part by the Key Research and Development Program of Shaanxi (No. 2020SF-148), the Science and Technology Supporting Xinjiang Project (No. 2020E0259), and partially supported by grants from the National Key Research and Development Program of China (No. 2017YFB1300303).

## Conflict of interest

The authors declare that the research was conducted in the absence of any commercial or financial relationships that could be construed as a potential conflict of interest.

## Publisher's note

All claims expressed in this article are solely those of the authors and do not necessarily represent those of their affiliated organizations, or those of the publisher, the editors and the reviewers. Any product that may be evaluated in this article, or claim that may be made by its manufacturer, is not guaranteed or endorsed by the publisher.
